# Lifestyle predictors for inconsistent participation to fecal based colorectal cancer screening

**DOI:** 10.1186/s12885-022-09287-9

**Published:** 2022-02-15

**Authors:** Markus Dines Knudsen, Ane Sørlie Kvaerner, Edoardo Botteri, Øyvind Holme, Anette Hjartåker, Mingyang Song, Espen Thiis-Evensen, Kristin Ranheim Randel, Geir Hoff, Paula Berstad

**Affiliations:** 1 Section for Colorectal Cancer Screening, Cancer Registry of Norway, P.O. Box 5313, 0304 Majorstuen, Oslo, Norway; 2 Department of Transplantation Medicine, Division of Surgery, Inflammatory Diseases and Transplantation, Norwegian PSC Research Center, Oslo University Hospital, P.O. Box 4950, 0424 Rikshospitalet, Nydalen, Oslo, Norway; 3 Departments of Epidemiology, Harvard T.H. Chan School of Public Health, 677 Huntington Avenue, 02115 Boston, MA USA; 4 Department of Research, Cancer Registry of Norway, P.O. Box 5313, 0304 Majorstuen, Oslo, Norway; 5 Department of Medicine, Sørlandet Hospital Kristiansand, P.O. Box 416, 4604 Lundsiden, Kristiansand, Norway; 6 Department of Health Management and Health Economis, Institute of Health and Society, University of Oslo, P.O. Box 1089, 0317 Blindern, Oslo, Norway; 7 Department of Nutrition, Institute of Basic Medical Sciences, University of Oslo, P.O. Box 1046, 0317 Blindern, Oslo, Norway; 8 Clinical and Translational Epidemiology Unit, Massachusetts General Hospital, Harvard Medical School, 55 Fruit Street, 02114 Boston, MA USA; 9 Division of Gastroenterology, Massachusetts General Hospital, Harvard Medical School, 55 Fruit Street, 02114 Boston, MA USA; 10 Department of Nutrition, Harvard T.H. Chan School of Public Health, MA Boston, USA; 11 Department of Research and Development, Telemark Hospital Trust, Ulefossvegen 55, 3710 Skien, Norway

## Abstract

**Background:**

Consistent participation in colorectal cancer (CRC) screening with repeated fecal immunochemical test (FIT) is important for the success of the screening program. We investigated whether lifestyle risk factors for CRC were related to inconsistent participation in up to four rounds of FIT-screening.

**Method:**

We included data from 3,051 individuals who participated in up to four FIT-screening rounds and returned a lifestyle questionnaire. Using logistic regression analyses, we estimated associations between smoking habits, body mass index (BMI), physical activity, alcohol consumption, diet and a healthy lifestyle score (from least favorable 0 to most favorable 5), and inconsistent participation (i.e. not participating in all rounds of eligible FIT screening invitations).

**Results:**

Altogether 721 (24%) individuals were categorized as inconsistent participants Current smoking and BMI ≥30 kg/m^2^ were associated with inconsistent participation; odds ratios (ORs) and 95% confidence intervals (CIs) were 1.54 (1.21-2.95) and 1.54 (1.20-1.97), respectively. A significant trend towards inconsistent participation by a lower healthy lifestyle score was observed (*p* < 0.05).

**Conclusions:**

Lifestyle behaviors were associated with inconsistent participation in FIT-screening. Initiatives aimed at increasing participation rates among those with the unhealthiest lifestyle have a potential to improve the efficiency of screening.

**Supplementary Information:**

The online version contains supplementary material available at 10.1186/s12885-022-09287-9.

## Introduction

Screening and adherence to a healthy lifestyle have both shown to decrease the incidence and mortality of colorectal cancer (CRC) [1, 2]. Smoking, overweight, physical inactivity, alcohol use, and low consumption of whole grains and dietary fiber are among the major modifiable risk factors related to early CRC death [3], but also to premature all-cause mortality in high-income countries [4]. The aim of CRC screening is to reduce CRC incidence and mortality. However, for a screening program to be successful the program requires an adequate participation rate, and that those who participate are individuals who will benefit from being screened [5, 6]. For fecal-based CRC screening programs, such as those using the fecal immunochemical test (FIT), this also means repeated participation over multiple screening rounds [7, 8]. Yet, less than 50% of the invited individuals consistently participate in all eligible screening rounds [9–12]. Identifying what characterizes these participants enables targeted interventions with the potential of improving participation rate and hence screening efficiency [5]. Research indicates that individuals with an unhealthy lifestyle participate to a lower degree in screening programs and other preventive activities [13, 14]. Indeed, we have previously shown that lifestyle risk factors for CRC were associated with non-participation in the second round of FIT screening after testing negative at the first round [15]. Similar results have been observed in other studies [16]. Nevertheless, there is limited knowledge whether these lifestyle factors also are associated with inconsistent participation in multiple CRC screening rounds. Assessing lifestyle behaviors at screening may help identify individuals at risk of inconsistent participation in FIT based CRC screening, individuals who potentially also have the highest risk of having neoplastic lesions.

## Methods

### Aims

The main aim of the study was to examine associations between proven lifestyle risk factors for colorectal neoplasia and inconsistent screening participation in up to four rounds of FIT in a longitudinal study. Further, we examined if the lifestyle risk factors were also associated with receiving a positive FIT result and detection of non-advanced or advanced lesions during up to four rounds of FIT based CRC screening.

### Study population

The present longitudinal study is a lifestyle sub-study within the ongoing Bowel Cancer Screening in Norway, a pilot study of a national CRC screening program. Bowel Cancer Screening in Norway is a comparative effectiveness trial that compares four biennial rounds of FIT with a single sigmoidoscopy. A total of 140 000 women and men aged 50-74 years (at the time of randomization) from two geographically defined areas in south-east Norway were randomly invited based on the population registry. The invitees were randomly assigned (1:1 ratio) to one of the two screening modalities. Individuals were included from 2012 to 2018. Individuals who died, moved out of the area, reached the upper age limit of 76 years for invitation, or received a CRC diagnosis before they were due for the first invitation were excluded from the trial [17].

For the present study, we included participants in the FIT arm who were invited to a lifestyle sub-study from November 2012 to September 2013 [18]. At first screening invitation, the invitees received a two-page lifestyle questionnaire (LSQ) along with a self-administrated home-based FIT kit. In order to be included in the present study, the LSQ had to be completed on paper or online before receiving the results of the corresponding FIT. Individuals were excluded from the present study at baseline if only returning the LSQ without returning any FIT tests. Furthermore, individuals with a positive FIT at the first round or only invited for first round were excluded.

**Fig. 1:**
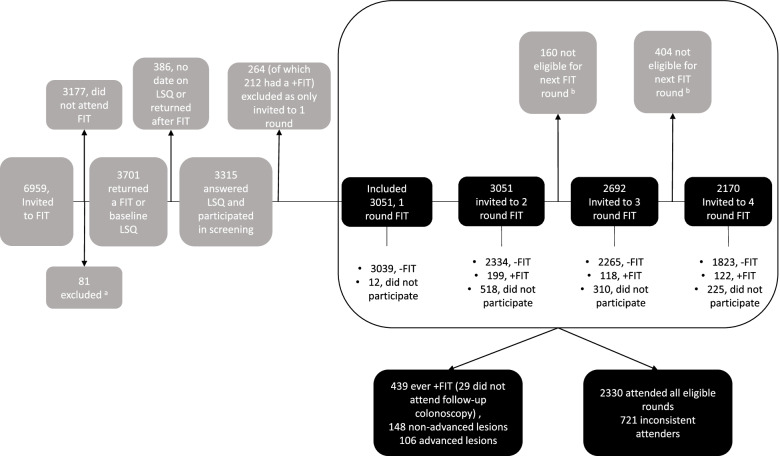
Flow chart of the study participants and inconsistent participation in up to four rounds of fecal immunochemical test (FIT) colorectal cancer screening and response to the lifestyle questionnaire (LSQ) at baseline in the lifestyle sub-study of the bowel cancer screening in Norway: a pilot study. Positive FIT = +FIT. Negative FIT = -FIT. ^a^ excluded due to dead, moved out of the area, previous colorectal cancer at baseline. ^b^ Not eligible for invitation for next round of FIT due to age ≥ 75, colorectal cancer diagnosis between FIT rounds, moved outside the county invited for screening, dead

By design, participants with a negative test result or no return of the FIT test were re-invited every second year, up to a maximum of four screening rounds. This practice was continued as long as the individual was eligible for participation. Individuals were no longer eligible for new FIT rounds at the time they reached the age of 76 years, died, moved out of the area included in the trial or being diagnosed with CRC between FIT rounds. Participants with a positive FIT result (15 µg hemoglobin/g feces) undergoing a follow-up colonoscopy were not eligible for any further FIT rounds [17]. See Fig. 1 for a flow chart of the study participants. Participants not returning the FIT sample within six weeks were sent a reminding letter, but no reminder for the LSQ was sent.

### Screening participation

Based on screening behavior, participants were classified into one of two categories: consistent screening participation (i.e. participants attending all eligible screening rounds invited to) and inconsistent screening participation (i.e. those not attending all eligible screening rounds they were invited to), see supplementary Table 1. Participants with a positive FIT were referred for a work-up colonoscopy. Based on the findings at the work-up colonoscopy, participants were categorized into the following categories: negative FIT screening and negative colonoscopy; no advanced lesions (non-advanced adenomas or serrated lesions < 10 mm and without dysplasia); advanced lesions (any adenoma ≥10 mm, villous component of at least 25% or high-grade dysplasia, advanced serrated lesion (including traditional serrated adenoma, hyperplastic polyps ≥10 mm or sessile serrated lesion with dysplasia or diameter ≥10 mm), or CRC [19].

### Lifestyle questionnaire

The two-page LSQ consisted of questions used in previous national surveys [20, 21] and the Norwegian Colorectal Cancer Prevention study (NORCCAP) [22] (supplementary Fig. 1). The participants were asked about demographic factors as well as lifestyle factors. Details about the LSQ and the included questions have been published previously [15]. Briefly, individuals were divided into four groups based on their smoking habits: Current, former <10 years since cessation, former ≥10 years since cessation and never smokers. Body mass index (BMI) was categorized according to international standards: <18.5 kg/m^2^, 18.5–24.9 kg/m^2^, 25.0–29.9 kg/m^2^ and ≥30.0 kg/m^2^ (obesity class I, II, III) [23]. Physical activity (times per week of 30 min of activity) was divided into quartiles. Alcohol consumption was divided into gender-specific quartiles, with non-drinkers as a fifth category. A dietary score was created based on quartiles of servings of (1) total fruit and vegetables (fruit, vegetables and berries), (2) total red and processed meat (steak, pork chops or similar, hamburgers or other dishes with minced meat, and sausages) and (3) fatty fish. Each of the dietary factors, 1-3, were divided into quartiles to create a dietary score (ranging from 0 to 3, 0 indicating the lowest consumption). One point was given to each of the following criteria: consumption of total red and processed meat in the first or second quartile; total fruit and vegetables in the third or fourth quartile; and fatty fish in the third or fourth quartile. Owing to the frequency categories in the LSQ, it was not possible to divide the variables into equally sized quartiles.

A healthy lifestyle score was created for each participant, assigning one point for complying to each of the following factors: never smoked or smoking cessation ≥10 years, BMI <25 kg/m^2^, physical activity of 30 min ≥7.0 times per week, low alcohol intake (women ≤7, men ≤14 glasses per week) and red and processed meat ≤4 times per week. One point was given if the consumption of fruit and vegetables was ≥3 per day and fatty fish was ≥1 time per week [24, 25]. The total number of points in the lifestyle score ranged from zero (poorest) to six (best). More detailed information on the lifestyle score has been published elsewhere [18].

### Statistical analyses

Baseline characteristics are presented as frequencies (numbers and percentages). Multivariable logistic regression analyses were used to examine the associations between the independent variables and the dichotomous outcome variables, that is, participation (consistent vs. inconsistent) and FIT result (negative vs. positive). Multinominal logistic regression were used to examine the associations between independent variables and the three levels of the outcome variable for lesions (negative screening vs. presence of non-advanced or advanced lesions). The logistic models were adjusted for the following covariates: age, sex, screening center, years of education, marital status, national background, smoking, BMI, physical activity, alcohol consumption, and the diet score. The statistical models where the lifestyle score was treated as the independent variable were adjusted for age, sex, screening center, years of education, marital status and national background.

For the analyses of inconsistent participation, sensitivity analyses, excluding participants with an ever positive FIT result, were carried out. Further, sensitivity analyses were done categorizing those participating in ≥3 rounds as consistent participants. Furthermore, we viewed the distribution of the most prominent lifestyle variables; smoking and BMI separately by educational lengths. We conducted supplementary analysis for inconsistent participation stratified by sex and age (<60/≥60 years).

Missing values were handled by using multiple imputation. We assumed that values were missing at random, as tests with missing data indicators did not show any associations to the outcome variables. Twenty datasets were imputed to reduce sampling variability from the imputation process [26]. We imputed missing variables used in the logistic regression models using the following variables: screening participation (consistent/inconsistent), FIT result (negative vs. positive), lesions (negative screening vs. presence of non-advanced or advanced lesions), center, age, and sex. We fitted separate logistic regression models, using a data available approach and analysis with missing as its own category in the variables as sensitivity analysis.

Analyses were performed using STATA™ software, version 17.1 (Stata Corp, College Station, Texas, USA).

## Results

Of the 6,959 individuals invited to answer the LSQ at first round of FIT screening, a total of 3,315 (48%) handed in the LSQ together with the FIT sample. Of these, 264 (8%) were invited to the baseline round only and were excluded (see Fig. 1). This resulted in 3,051 individuals of which 52% were women, being included in the analysis of inconsistent screening participation. 12% of the women and 17% of the men respondents returned the LSQ online. In age groups, the proportions of online-respondents were 15% and 13% for <60 and ≥ 60 years, respectively.

721 (24%) were categorized as inconsistent participants. Mean age at invitation was 60.8 and 59.8 y for consistent and inconsistent screening participants, respectively. Half of the inconsistent screening participants were female. The proportion of participants who participated at all screening rounds for which they were eligible, were 76% for those invited to 4 rounds, 73% for those invited to 3 rounds and 81% for those invited to 2 rounds (supplementary Table 1).

### Inconsistent participation

Lifestyle characteristics for consistent and inconsistent participants are shown in Table 1. Smoking behavior was inversely associated with inconsistent participation, p-trend < 0.01. Current smokers were most likely to be inconsistent participants vs. never smokers (OR 1.54, 95%CI 1.21-1.95). BMI was associated with being an inconsistent participant with a p-trend of < 0.01. Individuals with BMI ≥ 30 were most likely to be an inconsistent participants vs. BMI < 25 (OR 1.54, 95%CI 1.20-1.97). Alcohol intake was inversely associated with being an inconsistent participant, p-trend < 0.02. Individuals with alcohol intake in the second quartile vs. individuals in the first quartile were less likely to be an inconsistent participant (OR 0.67, 95%CI 0.49-0.91). An unhealthy lifestyle, measured by the lifestyle score, was associated with being an inconsistent participant, with a p-trend of 0.01. The stratified analysis for sex and age (<60/≥60 years) did not show any different association than observed in the main analysis (supplementary Tables 2 and supplementary Table 3).

**Table 1:** Lifestyle and diet characteristics for consistent and inconsistent –participation in up to four rounds of fecal immunochemical test (FIT), with adjusted odds ratio (OR) and 95% confidence intervals (CI)

Variables	Consistent participation, *n* = 2330, (% col)	Inconsistent participation, *n* = 721, (% col)	OR (95%CI) ^a^	*p*-value for trend^a^
Smoker				
Current	391 (17)	170 (24)	1.54 (1.21-1.95)	<0.01
Former ≤ 10year	315 (14)	95 (13)	0.80 (0.79-1.37)	
Former > 10year	670 (29)	184 (25)	0.99 (0.80-1.25)	
Never	947 (41)	271 (38)	Ref.	
Missing	7 (0)	1(0)		
Body Mass Index (kg m^-2^)				
16.9-24.9	991 (43)	278 (39)	Ref.	<0.01
25.0-29.9	995 (43)	285 (39)	0.96 (0.79-1.17)	
≥30.0	319 (14)	148 (20)	1.54 (1.20-1.97)	
Missing	25 (1)	10 (1)		
Physical activity 30min, times per week				
Q1 (≤1.5)	600 (26)	216 (30)	0.97 (0.76-1.24)	>0.05
Q2 (>1.5-≤3)	711 (31)	200 (28)	0.83 (0.65-1.06)	
Q3 (>2-≤6)	514 (22)	146 (20)	0.91 (0.70-1.19)	
Q4 (> 6)	486 (21)	153 (21)	Ref.	
Missing	19 (1)	6 (1)		
Alcohol, glasses per week				
Non-drinkers	387 (17)	161 (22)	1.13 (0.87-1.45)	0.02
Q1 ♀(>0-≤1.14), ♂(>0-≤2)	510 (22)	183 (25)	Ref.	
Q2 ♀(>1.14-≤2), ♂ (>2-≤3.8)	296 (13)	69 (10)	0.67 (0.49-0.91)	
Q3 ♀(>2-≤5), ♂ (>3.8-≤7.5)	506 (22)	144 (20)	0.79 (0.61-1.01)	
Q4 ♀(>5), ♂ (>7.5)	240 (10)	85 (12)	0.95 (0.70-1.29)	
Missing	391 (17)	79 (11)		
Diet score ^b^				
0	386 (17)	137 (19)	1.06 (0.73-1.55)	>0.05
1	972 (42)	296 (41)	0.95 (0.68-1.34)	
2	770 (33)	222 (31)	0.97 (0.69-1.37)	
3	186 (8)	53 (7)	Ref.	
Missing	16 (1)	13 (2)		
Healthy lifestyle score ^c^				
0-2	593 (26)	234 (32)	1.40 (0.98-2.01)	0.01
3	645 (28)	207 (29)	1.18 (0.82-1.69)	
4	464 (20)	128 (18)	1.07 (0.72-1.55)	
5-6	176 (8)	46 (6)	Ref.	
Missing	452 (19)	108 (15)		

^a^ Logistic regression analysis, adjusted for: age at first invitation (continues), sex, center, educational length, marital status, national background, smoking, body mass index, physical activity, alcohol, and diet score, was used to calculate OR and 95%CIs, multiple imputation was used for missing

^b^ Diet score: One point was given for each of the following criteria: consumption of total red and processed meat in the first or second quartile; total fruit and vegetables in the third or fourth quartile; and fatty fish in the third or fourth quartile

^c^ Score one point for each of the following factors, never smoked or smoking cessation ≥ 10 years, BMI (18.5-24.9), physical activity ≥ 7.0 times per week, alcohol intake (women ≤ 7, men ≤ 14 glass a week) and red and processed meat ≤ 4 times per week. One point was given if the consumption of fruit and vegetables was ≥ 3 per day and fatty fish was ≥ 1 per week. The logistic regression for the score was adjusted age at first invitation (continues), sex, center, educational length, marital status, national background

### Positive FIT

There were 439 (14%) with ever positive FIT, of whom 54% were male. Lifestyle characteristics for participants with and without a positive FIT are shown in Table 2.

**Table 2:** Lifestyle and diet characteristics for positive fecal immunochemical test (FIT) for up to four rounds of FIT, with adjusted odds ratio (OR) and 95% confidence intervals (CI)

Variables	Negative FIT, *n* = 2612, (% col)	Positive FIT, *n* = 439, (% col)	OR (95%CI)^a^	*p*-value for trend^a^
Smoker				
Current	461 (18)	100 (23)	1.58 (1.19-2.12)	<0.01
Former ≤ 10year	345 (13)	65 (15)	1.29 (0.93-1.79)	
Former > 10year	729 (28)	125 (29)	1.17 (0.90-1.52)	
Never	1070 (41)	148 (34)	Ref.	
Missing	7 (0)	1 (0)		
Body Mass Index (kg m^-2^)				
16.9-24.9	1103 (42)	166 (38)	Ref.	0.04
25.0-29.9	1094 (42)	186 (42)	1.06 (0.83-1.34)	
≥30.0	385 (15)	82 (19)	1.41 (1.04-1.91)	
Missing	30 (1)	5 (1)		
Physical activity 30min, times per week				
Q1 (≤1.5)	697 (25)	119 (27)	0.77 (0.57-1.03)	0.16
Q2 (>1.5-≤3)	781 (23)	130 (30)	0.80 (0.60-1.06)	
Q3 (>2-≤6)	580 (30)	80 (18)	0.70 (0.51-0.96)	
Q4 (> 6)	532 (20)	107 (24)	Ref.	
Missing	22 (1)	3 (1)		
Alcohol, glasses per week				
Non-drinkers	476 (18)	72 (16)	0.92 (0.65-1.28)	0.51
Q1 ♀(>0-≤1.14), ♂(>0-≤2)	593 (23)	100 (23)	Ref.	
Q2 ♀(>1.14-≤2), ♂ (>2-≤3.8)	300 (12)	65 (15)	1.29 (0.92-1.82)	
Q3 ♀(>2-≤5), ♂ (>3.8-≤7.5)	563 (22)	87 (20)	0.95 (0.69-1.31)	
Q4 ♀(>5), ♂ (>7.5)	273 (11)	52 (12)	1.11 (0.76-1.63)	
Missing	407 (16)	63 (14)		
Diet score ^b^				
0	443 (17)	80 (18)	0.96 (0.61-1.50)	0.93
1	1084 (42)	184 (42)	0.92 (0.61-1.38)	
2	856 (33)	136 (31)	0.93 (0.62-1.41)	
3	205 (8)	34 (8)	Ref.	
Missing	24 (1)	5 (1)		
Healthy lifestyle score^c^				
0-2	696 (27)	131 (30)	0.87 (0.57-1.32)	0.97
3	734 (28)	118 (27)	0.77 (0.50-1.16)	
4	513 (20)	79 (18)	0.78 (0.51-1.18)	
5-6	185 (7)	37 (8)	Ref.	
Missing	484 (19)	74 (17)		

^a^ Logistic regression analysis, adjusted for: age at first invitation (continues), sex, center, educational length, marital status, national background, smoking, body mass index, physical activity, alcohol, and diet score, was used to calculate OR and 95%CIs, multiple imputation was used for missing

^b^ Diet score: One point was given for each of the following criteria: consumption of total red and processed meat in the first or second quartile; total fruit and vegetables in the third or fourth quartile; and fatty fish in the third or fourth quartile

^c^ Score one point for each of the following factors, never smoked or smoking cessation ≥ 10 years, BMI (18.5-24.9), physical activity ≥ 7.0times per week, alcohol intake (women ≤ 7, men ≤ 14 glass a week) and red and processed meat ≤ 4 times per week. One point was given if the consumption of fruit and vegetables was ≥ 3 per day and fatty fish was ≥ 1 per week. The logistic regression for the score was adjusted age at first invitation (continues), sex, center, educational length, marital status, national background

Smoking was associated with having a positive FIT with a p-trend of < 0.01. The strongest association was observed for current smokers vs. never smokers (OR 1.58, 95%CI 1.19-2.12). BMI was associated with having a positive FIT with a p-trend of 0.04. The strongest association was observed for BMI ≥ 30 kg/m^2^ vs. BMI < 25 kg/m^2^ (OR 1.41, 95%CI 1.04-1.91). Individuals with physical activity in the third quartile vs. the fourths quartile were less likely to have a positive FIT (OR 0.70, 95%CI 0.51-0.96). However, no trend was observed.

### Advanced lesions

Of the 439 having a positive FIT, 410 had a follow-up colonoscopy. Of these, 148 (36%) were diagnosed with non-advanced lesions and 106 (26%) were diagnosed with advanced lesions. Table 3 shows lifestyle characteristics of the participants by colonoscopy result. Smoking was associated with being diagnosed with non-advanced and advanced -lesions with a p-trend of < 0.01 for both. The strongest associations were found for current smokers vs. never smokers; non-advanced lesions (OR 1.68, 95%CI 1.06-2.67) and advanced lesions (OR 2.54, 95%CI 1.48-4.36). BMI was associated with being diagnosed with non-advanced lesions (p-trend of 0.02), but not advanced lesions (p = 0.87). The strongest association was observed for BMI ≥ 30 vs. BMI < 25 (OR 1.76, 95%CI 1.08-2.89).

**Table 3:** Lifestyle and diet characteristics for negative fecal immunochemical test (FIT) test, non-advanced lesions and advanced lesions after a positive FIT for those attending the follow-up colposcopy result for up to four rounds of FIT, with adjusted odds ratio (OR) and 95% confidence intervals (CI)

Variables	Negative screening, *n* = 2768, (% col)	Non-advanced lesions, *n *= 148 (% col)	OR (95%CI)	*p*-value for trend ^a^	Advanced lesions, *n* = 106, (% col)	OR (95%CI) ^a^	*p*-value for trend ^a^
Smoker							
Current	486 (18)	35 (24)	1.68 (1.06-2.67)	0.01	31 (29)	2.54 (1.48-4.36)	<0.01
Former ≤ 10 year	367 (13)	28 (17)	1.50 (0.92-2.46)		13 (12)	1.24 (0.63-2.45)	
Former > 10 year	782 (28)	33 (22)	0.83 (0.53-1.32)		31 (29)	1.28 (0.76-2.16)	
Never	1125 (41)	52 (35)	Ref.		31 (29)	Ref.	
Missing	8 (0)	0 (0)			0 (0)		
Body Mass Index (kg m^−2^)							
16.9-24.9	1168 (42)	48 (32)	Ref.	0.02	43 (41)	Ref.	0.87
25.0-29.9	1150 (42)	69 (47)	1.33 (0.89-1.97)		49 (46)	1.16 (0.75-1.81)	
≥30.0	418 (15)	30 (20)	1.76 (1.08-2.89)		13 (12)	0.97 (0.50-1.87)	
Missing	32 (1)	1 (1)			1 (1)		
Physical activity 30 min, times per week							
Q1 (≤1.5)	739 (2277)	43 (29)	0.86 (0.53-1.40)	0.68	30 (28)	0.82 (0.47-1.43)	0.91
Q2 (>1.5-≤3)	826 (30)	43 (29)	0.87 (0.54-1.40)		34 (32)	0.87 (0.51-1.47)	
Q3 (>2-≤6)	614 (22)	28 (19)	0.81 (0.48-1.37)		15 (14)	0.51 (0.27-0.98)	
Q4 (> 6)	567 (21)	33 (22)	Ref.		27 (26)	Ref.	
Missing	22 (1)	1 (1)			0 (0)		
Alcohol, glasses per week							
Non-drinkers	501 (18)	25 (17)(	1.11 (0.62-2.00)	0.40	14 (13)	0.92 (0.46-1.84)	0.11
Q1 ♀(>0-≤1.14), ♂(>0-≤2)	635 (23)	30 (20)	Ref.		20 (19)	Ref.	
Q2 ♀(>1.14-≤2), ♂ (>2-≤3.8)	325 (12)	20 (14)	1.40 (0.79-2.49)		17 (16)	1.55 (0.79-3.05)	
Q3 ♀(>2-≤5), ♂ (>3.8-≤7.5)	590 (21)	30 (20)	1.14 (0.67-1.97)		23 (22)	1.12 (0.61-2.08)	
Q4 ♀(>5), ♂ (>7.5)	284 (10)	21 (14)	1.43 (0.79-2.58)		18 (17)	1.75 (0.88-2.49)	
Missing	433 (16)	22 (15)			14 (13)		
Diet score ^b^							
0	469 (17)	33 (22)	0.85 (0.43-1.67)	0.56	15 (14)	0.51 (0.22-1.15)	0.32
1	1156 (42)	50 (34)	0.56 (0.30-1.05)		49 (46)	0.66 (0.34-1.30)	
2	907 (33)	49 (33)	0.78 (0.42-1.45)		30 (28)	0.56 (0.28-1.13)	
3	211 (8)	14 (10)	Ref.		12 (11)	Ref.	
Missing	25 (1)	2 (1)			0 (0)		
Healthy lifestyle score ^c^							
0-2	732 (26)	55 (37)	1.28 (0.64-2.54)	0.17	32 (30)	0.82 (0.39-1.76)	0.90
3	781 (28)	34 (23)	0.80 (0.40-1.60)		30 (28)	0.71 (0.33-1.51)	
4	544 (20)	24 (16)	0.86 (0.41-1.79)		18 (17)	0.61 (0.28-1.34)	
5-6	197 (7)	10 (7)	Ref.		11 (10)	Ref.	
Missing	514 (19)	25 (17)			15 (14)		

^a^ Logistic regression analysis, adjusted for: age at first invitation (continues), sex, center, educational length, marital status, national background, smoking, body mass index, physical activity, alcohol, and diet score, was used to calculate OR and 95%CIs, multiple imputation was used for missing

^b^ Diet score: One point was given for each of the following criteria: consumption of total red and processed meat in the first or second quartile; total fruit and vegetables in the third or fourth quartile; and fatty fish in the third or fourth quartile

^c^ Score one point for each of the following factors, never smoked or smoking cessation ≥ 10 years, BMI (18.5-24.9), physical activity ≥ 7.0times per week, alcohol intake (women ≤ 7, men ≤ 14 glass a week) and red and processed meat ≤ 4 times per week. One point was given if the consumption of fruit and vegetables was ≥ 3 per day and fatty fish was ≥ 1 per week. The logistic regression for the score was adjusted age at first invitation (continues), sex, center, educational length, marital status, national background

In the two sensitivity analyses of inconsistent screening behavior, where only those with a negative FIT and those who had attended ≥ 3 out of 4 invitations were considered as consistent participants, similar results were observed (data not shown). Similarly, using alternative approaches for handling of missing data (i.e., a data available approach or treating missing as its own category) did not influence the results.

## Discussion

The current study represents a comprehensive analysis of lifestyle risk factors for CRC and inconsistent participation in up to four rounds of FIT based CRC screening. We found that smoking, BMI and an overall unhealthy lifestyle were associated with inconsistent participation. These factors were also associated with positive FIT and findings of lesions during the follow-up colonoscopy.

Since individuals with an unhealthy lifestyle are at increased risk of CRC [2], their screening participation is of particular interest. FIT screening is most efficient if individuals consistently attend at repeated screening rounds [7, 8]. To the best of our knowledge, this is the first long-term study to investigate lifestyle, inconsistency in FIT participation and screening results throughout four rounds of FIT based CRC screening. We have no information on how well the importance of consistent FIT testing is understood in the population. However, the proportion of participants not willing to repeat FIT screening after participating once was found to be less than 5% in the present pilot study [27]. That is, adherence to consistent participation in the population is remarkably lower than expected according to the attitudes towards FIT screening.

Previous studies have shown that unfavorable lifestyle factors such as smoking, overweight, low level of physical activity and low fruit and vegetables consumption have been associated with lack of participation in FIT screening [14, 16, 28]. Indeed, we have shown this for non-participation in the second round of FIT after participating in the first round [15].

Attendance in faecal-based screening has shown to be associated with socioeconomic status [29–33]. In our study, we observed smoking and obesity to be more prevalent in individuals with lower education than in individuals with higher education (data not shown). Despite this inequality in health behaviors by educational level, lifestyle factors were independently associated with both inconsistent participation and screening results after adjusting for education. This suggests that lifestyle risk factors for CRC are independent risk factors for inconsistent participation in FIT based CRC screening.

The present study has some important strengths, including the prospective design, the random invitation of individuals from the population registry, the large total sample size and that a large majority of first round screening participants completed the LSQ. Prevalence of current smokers in the present study (15%) was similar to the prevalence seen in the Norwegian population of similar age (15-20%) [34]. This suggests representative lifestyle characteristics, at least for smoking, in the study population. Furthermore, the usage of multiple imputation for missing data increased statistical power and reduced bias [35].

Several limitations of our study need to be noted, including the brevity of the LSQ and lack of validation. However, the specific questions included in the LSQ were copied from other validated questionnaires [20, 21]. The LSQ was rather short, designed to require less than 10 min to complete. This limited the possibility to analyze in detail dietary factors as a large number of questions are typically required to assess total dietary consumption. Furthermore, risk of confounding has to be considered. The population in the present study may be more homogenous in socioeconomic factors compared to population-based studies outside the Nordic countries [36]. Consequently, generalization to populations outside Norway should be done with caution. Studies have found a higher health consciousness to be related to screening participation [37]. Mental health has been associated with an unhealthy lifestyle [38] and high BMI has been associated with depression and poor mental health [39]. We have no information to clarify whether poor mental health explains the association between high BMI and inconsistent participation in the present study. The present study also lacks information on income and comorbidity to clarify whether the associations between unfavorable lifestyle characteristics and low screening participation can be explained by low socioeconomic status and comorbidity, both of which are predictors of low fecal screening uptake [30, 40]. Additionally, we had no information on family history of CRC or use of medications linked to CRC such as aspirin, which might have confounded the associations observed [2, 41].

Individually tailored CRC screening may contribute to a more cost-effective use of screening resources [42, 43]. This could mean once only endoscopy screening or more intensive FIT invitations for unhealthy individuals, while less intense FIT screening for healthy individuals. Certain subgroups of the population may be resistant to health messages such as cancer screening invitations, and targeted interventions are needed to encourage this subgroup to consistently participate in FIT screening, as also suggested by others [28]. Suitable interventions might include easy-to-read invitations which improve the understanding of screening aims and procedures in identified low-participation groups [44]. Personal recommendation from a trusted general practitioner might facilitate FIT participation of people with e.g. comorbidities related to high BMI [44]. Further studies should confirm the potential impact of smoking habits and BMI for tailored screening recommendations. Diet and physical activity are difficult to assess adequately in a screening setting. However, if valid tools for short assessment of these factors are available, their impact in personalized screening service should be investigated. Given the importance of lifestyle factors in the prevention of CRC, lifestyle registration may substantially improve ways to personalize CRC screening. The most optimal future CRC screening strategy is probably not a “one-size-fits-all’ approach.

## Conclusions

Our results indicate that inconsistent participation in FIT based CRC screening is associated with lifestyle behaviors linked to increased risk of positive FIT and detection of neoplastic lesions. Screening participants with unhealthy lifestyle characteristics might benefit from targeted invitations to comply repeated FIT screening.

## Supplementary Information

**Additional file 1.**

**Additional file 2.**

**Additional file 3.**

**Additional file 4.**

## Data Availability

Access to research data for external investigators, or use outside of the current protocol, will require approval from the Norwegian Regional Committee for Medical and Health Research Ethic and the Bowel cancer screening in Norway steering committee (information available on the project website: https://www.kreftregisteret.no/en/Research/Projects/personalizing-colorectal-cancer-screening-by-lifestyle/). Research data are not openly available because of the principles and conditions set out in articles 6[1] (e) and 9 [2] (j) of the General Data Protection Regulation (GDPR). Requests to access the datasets should be directed to the P.I Paula Berstad: pabe@kreftregisteret.no.
