# Supplementary material for: Lifestyle predictors for inconsistent participation to fecal based colorectal cancer screening
Source: BMC Cancer. 2022 Feb 15;22:172. doi: 10.1186/s12885-022-09287-9 (PMC8848967; doi:10.1186/s12885-022-09287-9)
Supplement: Supplementary file 1 — Additional file 1. [file 12885_2022_9287_MOESM1_ESM.pdf]

Evaluation: Questionnaire on lifestyle

We kindly ask you to consider replying to some questions about lifestyle in the context of screening for colorectal cancer. Your answers are important, whether you are invited to the screening examination itself or not (i.e. you are in the ”control group”) and whether you decide to participate or not in the screening examination.

You can preferably complete the questionnaire online at www.tarmkreftscreening.no below ”Evaluation”. To log on you can use BankID, Buypass or Commfides. You may order PIN-code and password first time you log on if needed. Remember possible reward (price draw) for those filling in the questionnaire online. If you do not wish to use internet, you can use this paper questionnaire and return it in the reply envelope.

Every question should be answered by marking the box that fits you best with an X or a number. If you are unsure, please, answer as well as you can. An estimate is better than no answer.

Date for completion: Day ☐☐ Month ☐☐ Year 20☐☐

1. PERSONAL DETAILS

1.1 WEIGHT: *(kilograms)* ☐☐☐ kg **HEIGHT** *(cm)* ☐☐☐ cm

1.2 Your national background (your parent's place of birth)
(only one mark)
If your parents have different countries of birth, mark the country/region to which you relate most closely.

- ☐ Norway
- ☐ Northern or Central Europe outside Norway, North America, Australia
- ☐ Southern Europe, South- or Central America
- ☐ Asia
- ☐ Africa

1.3 Marital status *(only one mark)*

- ☐ Single
- ☐ Married/ cohabiting
- ☐ Widow(er)
- ☐ Divorced/ separated

1.4 Mark the highest level of completed education

- ☐ Primary school
- ☐ High school
- ☐ University/ college *(at least 2 years completed)*

2. OCCUPATION

2.1 Are you for the time being: *(only one mark)*

- ☐ Working
- ☐ Retired
- ☐ Homemaker
- ☐ Unemployed
- ☐ Disabled/ on rehabilitation/ on long term sick leave *(more than 3 months)*
- ☐ On disability pension, possibly combined with working or other financial support *(e.g. retirement pension)*

3. SMOKING

3.1 Do you currently smoke? *(only one mark)*

- ☐ Yes, daily
- ☐ Yes, occasionally
- ☐ Have quitted
- ☐ Have never smoked

Smoked earlier, but quitted ☐☐ years ago

3.2 Are you smoking other than cigarettes daily?

- ☐ Yes, pipe
- ☐ Yes, cigar/ cigarillos

3.3 If you are a current daily smoker, how many do you smoke daily?

- ☐ 1-5
- ☐ 6-10
- ☐ 11-20
- ☐ More than 20

4. EXERCISE (walking, cycling, jogging, swimming etc.)

4.1 Have you, during the last three years suffered from a chronic disease or pain restricting your physical activity?
(e.g. hip ailments or cardiovascular disease)

- ☐ Yes
- ☐ No

4.2 How often do you exercise at least 30 minutes *<u>without</u>* sweating or shortness of breath? *(only one mark)*

Never	Less than once a week	1-2 times/ a week	3-4 times/ a week	5-7 times/ a week	more than 7 times/ a week
☐	☐	☐	☐	☐	☐

4.3 How often do you exercise at least 30 minutes *<u>so that you</u>* sweat or get short of breath? *(only one mark)*

Never	Less than once a week	1-2 times/ a week	3-4 times/ a week	5-7 times/ a week	more than 7 times/ a week
☐	☐	☐	☐	☐	☐

Please, continue on next page

5. FOOD AND DRINK

5.1 How often do you usually eat these foods? *(only one mark per line) (t. = times)*

	Seldom/ never	1-3 t./ month	1-3 t./ week	4-6 t./ week	1-2 t./ day	3 t. or more/day
Fruit, berries	☐	☐	☐	☐	☐	☐
Raw vegetables, salad	☐	☐	☐	☐	☐	☐
Boiled vegetables....	☐	☐	☐	☐	☐	☐
Boiled potatoes.......	☐	☐	☐	☐	☐	☐
Cheese (all types)...	☐	☐	☐	☐	☐	☐
Poultry for dinner…. *(e.g. chicken, turkey)*	☐	☐	☐	☐	☐	☐
Steak, pork chops or similar for dinner..	☐	☐	☐	☐	☐	☐
Hamburger or other dishes with minced meat for dinner…….	☐	☐	☐	☐	☐	☐
Sausages or dishes with sausages for dinner………………	☐	☐	☐	☐	☐	☐
Fatty fish for dinner or on sandwich........ *(e.g. salmon, trout, mackerel, herring)*	☐	☐	☐	☐	☐	☐
Chocolate, candy….	☐	☐	☐	☐	☐	☐
Potato crisps............	☐	☐	☐	☐	☐	☐

5.2 How many slices of bread do you usually eat per day? *(only one mark per line)*

**Slices per day:**	Do not use	Less than 1	1-2	3-4	5-6	7+
White bread……….	☐	☐	☐	☐	☐	☐
Partly-refined bread	☐	☐	☐	☐	☐	☐
Brown bread……… *(incl. crispy bread)*	☐	☐	☐	☐	☐	☐

5.3 How many glasses of milk (sweet or sour) do you usually drink per. day? *(only one mark per line)*

**Glasses per day:**	Do not use	less than 1	1-2	3-4	5 +
Whole fat milk……..	☐	☐	☐	☐	☐
Semi-fat milk ……..	☐	☐	☐	☐	☐
Skimmed or “extra light” milk…...	☐	☐	☐	☐	☐

5.4 What type of fat do you usually use?
(one mark per. column)

	on bread	in cooking
Butter ……………………………	☐	☐
Butter-oil mixture……………….. *(e.g. Bremykt or Brelett)*	☐	☐
Melange………………………….	☐	☐
Soft/light margarine……………..	☐	☐
Oils ………………………………	☐	☐
Do not use fat……………………	☐	☐

5.5 How often have you consumed alcohol during the last year? *(light and alcohol free beer not to be included)*

- Never ……………………………………….. ☐
- Have not consumed alcohol during the last year ………………………… ☐
- A few times only during the last year…….. ☐
- Approximately once a month……………… ☐
- 2-3 times a month…………………….…. ☐
- once a week ……………………………….. ☐
- 2-3 times a week…………………………… ☐
- 4-7 times a week…………………………… ☐

For those who have consumed alcohol during the last year:

5.6 When drinking alcohol, how many glasses and/or drinks do you usually have?

Number of glasses ☐☐

5.7 Approximately how many times during the last year have you consumed more than 5 glasses and/or drinks within 24 hours?

Number of times ☐☐

5.8 When you drink alcohol, do you usually drink:
(Mark as many as you want)

☐ Beer ☐ Wine ☐ Spirits

6. NUTRITIONAL SUPPLEMENTS

6.1 Do you use any of these supplements?

	Daily	Occasionally	No
Cod-liver oil/ Cod-liver oil capsules/ fish oil-capsules/ omega 3 fatty acids………….....	☐	☐	☐
Vitamin D-/ multi vitamins containing vitamin D…...........	☐	☐	☐

Thank you for your help!
Please, return in the enclosed envelope marked ”Evaluation”.
