# Supplementary material for: Lifestyle predictors for inconsistent participation to fecal based colorectal cancer screening
Source: BMC Cancer. 2022 Feb 15;22:172. doi: 10.1186/s12885-022-09287-9 (PMC8848967; doi:10.1186/s12885-022-09287-9)
Supplement: Supplementary file 2 — Additional file 2. [file 12885_2022_9287_MOESM2_ESM.docx]

| Supplementary table 1. Number of times attended fecal immunochemical test (FIT) screening according to number of rounds invited, of those who returned the baseline lifestyle questionnaire. Consistent participates, illustrated by the gray shading. | | | | | |
| --- | --- | --- | --- | --- | --- |
|  | Rounds invited, n (%col) | | | |  |
|  | 1, n = 0 | 2, n = 359 | 3, n = 522 | 4, n = 2170 | Total |
| Rounds attended |  |  |  |  |  |
| 1 | - | 68 (19) | 45 (9) | 91 (4) | 204 (7) |
| 2 | - | 291 (81) | 93 (18) | 127 (6) | 511 (17) |
| 3 | - | - | 384 (73) | 297 (14) | 681 (22) |
| 4 | - | - | - | 1655 (76) | 1655(54) |
