# Supplementary material for: Lifestyle predictors for inconsistent participation to fecal based colorectal cancer screening
Source: BMC Cancer. 2022 Feb 15;22:172. doi: 10.1186/s12885-022-09287-9 (PMC8848967; doi:10.1186/s12885-022-09287-9)
Supplement: Supplementary file 3 — Additional file 3. [file 12885_2022_9287_MOESM3_ESM.docx]

| Supplementary table 2. Lifestyle and diet characteristics for consistent and inconsistent –participation in up to four rounds of fecal immunochemical test (FIT), with adjusted odds ratio (OR) and 95% confidence intervals (CI) by sex. | | | | | | | | | | | | | | |
| --- | --- | --- | --- | --- | --- | --- | --- | --- | --- | --- | --- | --- | --- | --- |
|  | Women | | | | | | | | Men | | | | | |
| Variables | Consistent participation, n=1257, (% col) | | | Inconsistent participation, n=359, (% col) | OR (95%CI) ^a^ | | | p-value for trend^a^ | Consistent participation, n=,1073 (% col) | | | Inconsistent participation, n=362, (% col) | OR (95%CI) ^a^ | p-value for trend^a^ |
| Smoker | |  |  | | |  |  | | |  |  |  |  |  |
| Current | 226 (18) | | | 93 (26) | 1.69 (1.22, 2.35) | | | <0.01 | 165 (15) | | | 77 (21) | 1.41 (0.99, 2.00) | 0.05 |
| Former ≤ 10year | 155 (12) | | | 44 (12) | 1.13 (0.76, 1.69) | | |  | 160 (15) | | | 51 (14) | 0.97 (0.66, 1.43) |  |
| Former > 10year | 334 (27) | | | 84 (23) | 1.06 (0.77, 1.69) | | |  | 336 (31) | | | 100 (28) | 1.00 (0.73, 1.37) |  |
| Never | 537 (43) | | | 137 (38) | Ref. | | |  | 410 (38) | | | 134 (37) | Ref. |  |
| Missing | 5 (0) | | | 1(0) |  | | |  | 2 (0) | | | 0 (0) |  |  |
| Body Mass Index (kg m^-2^) | | | | | | | | |  | | |  |  |  |
| 16.9-24.9 | 659 (52) | | | 168 (47) | Ref. | | | <0.01 | 332 (31) | | | 110 (30) | Ref. | >0.05 |
| 25.0-29.9 | 421 (34) | | | 104 (29) | 1.02 (0.77, 1.35) | | |  | 574 (54) | | | 181 850) | 0.93 (0.70, 1.23) |  |
| ≥30.0 | 163 (13) | | | 79 (22) | 1.95 (1.38, 2.74) | | |  | 156 (15) | | | 69 (19) | 1.25 (0.86, 1.82) |  |
| Missing | 14 (1) | | | 8 (2) |  | | |  | 11 (1) | | | 2 (0) |  |  |
| Physical activity 30min,  times per week | | | | | | | | |  | | |  |  |  |
| Q1 (≤1.5) | 281 (22) | | | 91 (25) | 1.04 (0.72,1.49) | | | >0.05 | 319 (30) | | | 125 (35) | 0.90 (0.63, 1.27) | >0.05 |
| Q2 (>1.5-≤3) | 381 (30) | | | 109 (30) | 1.01 (0.72, 1.42) | | |  | 330 (31) | | | 91 (25) | 0.67 (0.47, 0.96) |  |
| Q3 (>2-≤6) | 299 (24) | | | 83 (23) | 1.07 (0.75, 1.53) | | |  | 215 (20) | | | 63 (17) | 0.76 (0.51, 1.12) |  |
| Q4 (> 6) | 285 (23) | | | 75 (21) | Ref. | | |  | 201 (19) | | | 78 (22) | Ref. |  |
| Missing | 11 (1) | | | 1 (0) |  | | |  | 8 (1) | | | 5 (1) |  |  |
| Alcohol, glasses per week | | | | | | | | |  | | |  |  |  |
| Non-drinkers | 260 (21) | | | 98 (27) | 1.14 (0.78, 1.64) | | | >0.05 | 127 (12) | | | 63 (17) | 1.19 (0.81, 1.73) | 0.04 |
| Q1 ♀(>0-≤1.14), ♂(>0-≤2) | 253 (20) | | | 78 (22) | Ref. | | |  | 257 (24) | | | 105 (29) | Ref. |  |
| Q2 ♀(>1.14-≤2), ♂ (>2-≤3.8) | 185 (15) | | | 37 (10) | 0.66 (0.43, 1.03) | | |  | 111 (10) | | | 32 (9) | 0.73 (0.46, 1.16) |  |
| Q3 ♀(>2-≤5), ♂ (>3.8-≤7.5) | 257 (20) | | | 73 (20) | 0.92 (0.63, 1.34) | | |  | 249 (23) | | | 71 (20) | 0.69 (0.48, 0.98) |  |
| Q4 ♀(>5), ♂ (>7.5) | 119 (10) | | | 37 (10) | 1.00 (0.63, 1.59) | | |  | 121 (11) | | | 48 (13) | 0.91 (0.59, 1.40) |  |
| Missing | 183 (15) | | | 36 (10) |  | | |  | 208 (19) | | | 43 (12) |  |  |
| Diet score ^b^ | | | | | | | | |  | | |  |  |  |
| 0 | 135 (11) | | | 49 (19) | 1.26 (0.75, 2.13) | | | >0.05 | 251 (23) | | | 88 (24) | 0.88 (0.48, 1.58) | >0.05 |
| 1 | 481 (38) | | | 134 (41) | 1.00 (0.65, 1.56) | | |  | 491 (46) | | | 162 (45) | 0.85 (0.48, 1.49) |  |
| 2 | 499 (40) | | | 136 (31) | 1.02 (0.66, 1.57) | | |  | 271 (25) | | | 86 (24) | 0.84 (0.47, 1.52) |  |
| 3 | 131 (10) | | | 34 (7) | Ref. | | |  | 55 (5) | | | 19 (5) | Ref. |  |
| Missing | 11 (1) | | | 6 (2) |  | | |  | 5 (1) | | | 7(2) |  |  |
| Healthy lifestyle score ^c^ |  | | |  |  | | |  |  | | |  |  |  |
| 0-2 | 264 (21) | | | 106 (30) | 1.86 (1.17,2.97) | | | <0.01 | 329 (31) | | | 128 (35) | 1.01 (0.57, 1.80) | >0.05 |
| 3 | 336 (27) | | | 103 (29) | 1.48 (0.93, 2.34) | | |  | 309 (29) | | | 104 (29) | 0.88 (0.49,1.59) |  |
| 4 | 306 (24) | | | 70 (20) | 1.09 (0.67, 1.76) | | |  | 158 (15) | | | 58 (16) | 1.03 (0.55,1.94) |  |
| 5-6 | 130 (10) | | | 29 (8) | Ref. | | |  | 46 (4) | | | 17 (5) | Ref. |  |
| Missing | 221 (18) | | | 51 (14) |  | | |  | 231 (22) | | | 55 (15) |  |  |

^a^ Logistic regression analysis, adjusted for: age at first invitation (continues), sex, center, educational length, marital status, national background, smoking, body mass index, physical activity, alcohol, and diet score, was used to calculate OR and 95%CIs, multiple imputation was used for missing.
^b^ Diet score: One point was given for each of the following criteria: consumption of total red and processed meat in the first or second quartile; total fruit and vegetables in the third or fourth quartile; and fatty fish in the third or fourth quartile.
^c^ Score one point for each of the following factors, never smoked or smoking cessation ≥ 10 years, BMI (18.5-24.9), physical activity ≥ 7.0 times per week, alcohol intake (women ≤ 7, men ≤ 14 glass a week) and red and processed meat ≤ 4 times per week. One point was given if the consumption of fruit and vegetables was ≥ 3 per day and fatty fish was ≥ 1 per week. The logistic regression for the score was adjusted age at first invitation (continues), sex, center, educational length, marital status, national background.
