# Supplementary material for: Lifestyle predictors for inconsistent participation to fecal based colorectal cancer screening
Source: BMC Cancer. 2022 Feb 15;22:172. doi: 10.1186/s12885-022-09287-9 (PMC8848967; doi:10.1186/s12885-022-09287-9)
Supplement: Supplementary file 4 — Additional file 4. [file 12885_2022_9287_MOESM4_ESM.docx]

| Supplementary table 3. Lifestyle and diet characteristics for consistent and inconsistent –participation in up to four rounds of fecal immunochemical test (FIT), with adjusted odds ratio (OR) and 95% confidence intervals (CI) by age group. | | | | | | | | | | | | | | |
| --- | --- | --- | --- | --- | --- | --- | --- | --- | --- | --- | --- | --- | --- | --- |
|  | Age <60 | | | | | | | | Age ≥ 60 | | | | | |
| Variables | Consistent participation, n=1105, (% col) | | | Inconsistent participation, n=392, (% col) | OR (95%CI) ^a^ | | | p-value for trend^a^ | Consistent participation, n=,1225 (% col) | | | Inconsistent participation, n=329, (% col) | OR (95%CI) ^a^ | p-value for trend^a^ |
| Smoker | |  |  | | |  |  | | |  |  |  |  |  |
| Current | 227 (21) | | | 109 (28) | 1.67 (1.22, 2.30) | | | <0.01 | 164 (13) | | | 61 (19) | 1.49 (1.03, 2.17) | 0.05 |
| Former ≤ 10year | 154 (14) | | | 54 (14) | 1.14 (0.78, 1.66) | | |  | 161 (13) | | | 41 (14) | 0.98 (0.65, 1.48) |  |
| Former > 10year | 262 (24) | | | 75 (19) | 0.93 (0.67, 1.30) | | |  | 408 (33) | | | 109 (33) | 1.11 (0.82, 1.50) |  |
| Never | 459 (42) | | | 153 (39) | Ref. | | |  | 488 (40) | | | 118 (36) | Ref. |  |
| Missing | 3 (0) | | | 1 (0) |  | | |  | 4 (0) | | | 0 (0) |  |  |
| Body Mass Index (kg m^-2^) | | | | | | | | |  | | |  |  |  |
| 16.9-24.9 | 464 (42) | | | 152 (47) | Ref. | | | >0.05 | 527 (43) | | | 126 (38) | Ref. | 0.01 |
| 25.0-29.9 | 470 (43) | | | 160 (29) | 1.02 (0.77, 1.33) | | |  | 525 (43) | | | 125 (38) | 0.95 (0.71, 1.26) |  |
| ≥30.0 | 164 (15) | | | 76 (22) | 1.44 (1.02, 2.05) | | |  | 155 (13) | | | 72 (22) | 1.77 (1.24,2.54) |  |
| Missing | 7 (1) | | | 4 (2) |  | | |  | 18 (2) | | | 6 (2) |  |  |
| Physical activity 30min,  times per week | | | | | | | | |  | | |  |  |  |
| Q1 (≤1.5) | 288 (26) | | | 112 (29) | 0.89 (0.63, 1.26) | | | >0.05 | 312 (26) | | | 104 (32) | 1.06 (0.74, 1.53) | >0.05 |
| Q2 (>1.5-≤3) | 355 (32) | | | 111 (28) | 0.77 (0.55, 1.08) | | |  | 356 (29) | | | 89 (27) | 0.89 (0.62, 1.29) |  |
| Q3 (>2-≤6) | 223 (20) | | | 79 (20) | 0.91 (0.63, 1.31) | | |  | 291 (24) | | | 67 (20) | 0.89 (0.61, 1.31) |  |
| Q4 (> 6) | 228 (21) | | | 86 (22) | Ref. | | |  | 258 (21) | | | 67 (20) | Ref. |  |
| Missing | 11 (1) | | | 4 (1) |  | | |  | 8 (1) | | | 2 (1) |  |  |
| Alcohol, glasses per week | | | | | | | | |  | | |  |  |  |
| Non-drinkers | 163 (21) | | | 75 (19) | 1.01 (0.70, 1.45) | | | >0.05 | 224 (18) | | | 86 (26) | 1.28 (0.90, 1.83) | >0.05 |
| Q1 ♀(>0-≤1.14), ♂(>0-≤2) | 226 (20) | | | 101 (26) | Ref. | | |  | 284 (23) | | | 82 (25) | Ref. |  |
| Q2 ♀(>1.14-≤2), ♂ (>2-≤3.8) | 149 (15) | | | 43 (11) | 0.71 (0.47, 1.08) | | |  | 147 (12) | | | 26 (8) | 0.64 (0.39, 1.04) |  |
| Q3 ♀(>2-≤5), ♂ (>3.8-≤7.5) | 259 (20) | | | 81 (21) | 0.71 (0.50,1.01) | | |  | 247 (20) | | | 63 (19) | 0.90 (0.62, 1.31) |  |
| Q4 ♀(>5), ♂ (>7.5) | 108 (10) | | | 46 (12) | 0.88 (0.57, 1.37) | | |  | 132 (11) | | | 39 (12) | 1.04 (0.67, 1.63) |  |
| Missing | 200 (18) | | | 46 (12) |  | | |  | 191 (16) | | | 33 (10) |  |  |
| Diet score ^b^ | | | | | | | | |  | | |  |  |  |
| 0 | 215 (20) | | | 80 (20) | 0.87 (0.52, 1.46) | | | >0.05 | 171 (14) | | | 57 (17) | 1.34 (0.76, 2.36) | >0.05 |
| 1 | 464 (42) | | | 161 (41) | 0.81 (0.51, 1.31) | | |  | 508 (42) | | | 135 (41) | 1.08 (0.65, 1.80) |  |
| 2 | 347 (31) | | | 116 (30) | 0.79 (0.49, 1.29) | | |  | 423 (35) | | | 106 (32) | 1.11 (0.70, 1.85) |  |
| 3 | 74 (8) | | | 30 (8) | Ref. | | |  | 112 (9) | | | 23 (7) | Ref. |  |
| Missing | 5 (1) | | | 5 (1) |  | | |  | 11 (1) | | | 8 (2) |  |  |
| Healthy lifestyle score ^c^ |  | | |  |  | | |  |  | | |  |  |  |
| 0-2 | 317 (29) | | | 139 (36) | 1.71 (1.01, 2.87) | | | 0.05 | 276 (23) | | | 95 (29) | 1.25 (0.74, 2.12) | >0.05 |
| 3 | 288 (26) | | | 102 (26) | 1.35 (0.80, 2.27) | | |  | 357 (29) | | | 105 (32) | 1.08 (0.65, 1.80) |  |
| 4 | 194 (18) | | | 72 (18) | 1.42 (0.83, 2.45) | | |  | 270 (22) | | | 56 (17) | 0.83 (0.48, 1.44) |  |
| 5-6 | 82 (7) | | | 21 (5) | Ref. | | |  | 94 (8) | | | 25 (8) | Ref. |  |
| Missing | 224 (20) | | | 58 (15) |  | | |  | 228 (19) | | | 48 (15) |  |  |

^a^ Logistic regression analysis, adjusted for: age at first invitation (continues), sex, center, educational length, marital status, national background, smoking, body mass index, physical activity, alcohol, and diet score, was used to calculate OR and 95%CIs, multiple imputation was used for missing.
^b^ Diet score: One point was given for each of the following criteria: consumption of total red and processed meat in the first or second quartile; total fruit and vegetables in the third or fourth quartile; and fatty fish in the third or fourth quartile.
^c^ Score one point for each of the following factors, never smoked or smoking cessation ≥ 10 years, BMI (18.5-24.9), physical activity ≥ 7.0 times per week, alcohol intake (women ≤ 7, men ≤ 14 glass a week) and red and processed meat ≤ 4 times per week. One point was given if the consumption of fruit and vegetables was ≥ 3 per day and fatty fish was ≥ 1 per week. The logistic regression for the score was adjusted age at first invitation (continues), sex, center, educational length, marital status, national background.
